# Caregiver Knowledge About Palliative Care in King Abdullah Medical City, Makkah, Saudi Arabia: A Cross-Sectional Study (2023)

**DOI:** 10.7759/cureus.50258

**Published:** 2023-12-10

**Authors:** Alaa S Bakhsh, Rami I Faraj, Mohammed Alashqar, Imtinan Alsahafi

**Affiliations:** 1 Palliative Care, King Abdullah Medical City, Makkah, SAU; 2 Family Medicine, King Abdulaziz Medical City, Jeddah, SAU

**Keywords:** palliative care education, awareness, knowledge, caregiver, palliative care

## Abstract

Background

Understanding palliative care (PC) can hinder access to it. To address this, further research into the factors influencing knowledge and awareness of PC is required to develop effective public health campaigns. This study aimed to estimate the knowledge and awareness of PC among primary family caregivers of patients present to the palliative department at King Abdullah Medical City, Makkah, Kingdom of Saudi Arabia (KSA), in 2023, and to determine the factors affecting the family caregiver's awareness about PC.

Materials and methods

This cross-sectional study was conducted in 2023 among patients' family caregivers in the palliative department of King Abdullah Medical City, Makkah, KSA. The patient was evaluated by palliative service as an inpatient or outpatient presentation. A score, namely "caregivers' general knowledge of palliative care," was used as a study variable. Each of the questions was converted to points and calculated using a simple additive method. The total score was converted to a categorical variable. To discard the null hypothesis, a conventional p-value <0.05 was used.

Results

The 378 family primary caregivers completed the self-administered questionnaire. The majority (73.8%) were unaware of PC until their family member was admitted to the hospital. The mean score of caregivers’ general knowledge is 2.75+0.9, indicating that they have limited knowledge about PC. Age, employment status, relation to the patient, and duration of receiving PC are significantly associated with their knowledge. No associations were found on gender, level of education, and tumor site. Participants aged 26-35 years old (p<0.001), those who are students (p=0.002), who are brother/sister of the patient (p<0.001), and less than one year of PC (p<0.001) significantly related to caregivers’ knowledge.

Conclusion

A low knowledge mean score was found in this study. This indicated that participants had limited knowledge of PC. This study suggested that effective PC education programs and increasing specialized facilities be developed to raise the awareness of both caregivers and patients.

## Introduction

Palliative care (PC) is a medical specialty that focuses on improving the quality of life while addressing physical symptoms, mental health, social issues, and spiritual well-being of patients and their families diagnosed with life-threatening diseases [1-3].

The benefits of PC include the prevention and relief from discomfort by early identification and management of the disease. Moreover, it gives a network of support to help the patient's family and caregivers manage the disease. The inclusion of PC into the healthcare system increases satisfaction among patients and improves well-being [3-5]. In terms of statistics, roughly 56.8 million individuals are diagnosed with life-threatening diseases each year, with 25.7 million requiring PC for themselves or their families [3].

Despite the benefits mentioned earlier, the utilization of PC is not maximized. Studies have shown that one possible reason for the underutilization of PC is poor or low knowledge of PC [6]. Publications revealed poor knowledge of PC by family members, as caregivers perceive PC as a label of hopelessness and despair. One of these studies found that half of caregivers for terminally ill patients never understand the concept of PC [7,8].

As PC has grown significantly since the Ministry of Health included the last palliative phase as part of the Kingdom of Saudi Arabia (KSA)'s 2030 transformation plan, caregivers and family members must gain a thorough understanding of this specialty to evaluate any deficiencies that may undermine the patient's advanced directive plan [9].

Family caregivers are considered a very significant element and central consumers of PC and hospice because they will be a part of the palliative goals of care at some point. Thus, our rationale is to focus on these groups [10].

The authors selected this topic based on multiple situations encountered in actual clinical practice, which concluded that poor understanding by a caregiver of the specialty leads to complicated grief behavior, unacceptance of expected clinical decline in function, and various consumption of medical services for stabilized palliative case condition, so the researcher rationale has been built to study essential background for the primary caregiver that may affect patients management [11].

This study aimed to estimate the knowledge of PC among primary family caregivers in patients present to the PC department at King Abdullah Medical City (KAMC), Makkah, KSA, in 2023, and to do multiple correlations to determine the factors affecting the family caregiver awareness of PC.

## Materials and methods

This was a single-center cross-sectional study of patients' family caregivers following with the Palliative Department of KAMC, Makkah, KSA, which was conducted from Jan 2023 till Aug 2023.

PC service at KAMC consists of inpatient, outpatient, and consultations. The inpatient admission unit consists of 10 beds, while the consultation service coverage may reach 25 cases per day, either as a new consultation or follow-up. In the outpatient, there are three clinics per week, with an average of 10 patients scheduled in each clinic. As KAMC is an oncology center, our sample was made up of oncology cases. The sample size has been calculated via Raosoft (Raosoft Inc., Seattle, Washington) based on the number of palliative patients in KAMC, and given the recommended 377 samples, the estimated time to reach the target was between four and six months.

The inclusion criteria of participants involved were above the age of 18, from both male and female genders, and they were family caregivers of an oncology and non-oncology patient who was treated for palliative intent either as an inpatient or outpatient service. On the other hand, the exclusion criteria included family caregivers of oncology patients who were still receiving curative treatment, the age of caregivers less than 18 years old, palliative patients who were referred to other health institutions, and refusal to consent.

Data collection

Data were collected from the family caregiver via printed paper without any nominative information. Participants were interviewed using a structured questionnaire. A serial study code was used to identify each participant. These were linked to the patient’s name and medical record number (MRN) in a separate identification log sheet that was kept in a safe, locked place.

The study questionnaire was taken after approval from the Jazan team; the questionnaire was originally based on the semi-structured interview protocol developed by McIlfatrick et al. [12]. The first part of the questionnaire focused on the caregivers’ socio-demographic characteristics (age, gender, educational level, job, and relationship to the patient), as well as the patient’s cancer type and the duration of follow-up with the PC unit. The subsequent three sections aimed to elicit (1) their knowledge and perceptions of PC, (2) their expectations and accessibility for PC service provision, and (3) their suggestions for future strategies to promote awareness of PC (as shown in Appendix 1). Each item scored 1 point, and then the total score was converted to a categorical variable. The score was categorized as follows: caregivers’ general knowledge of PC not aware (<2), slightly aware (<4-2), somewhat aware (<6-4), and very aware (6-8).

The data from this research were evaluated with Statistical Product and Service Solutions (SPSS, version 27) (IBM Corp., Armonk, NY) and graphically presented with GraphPad Prism version 8 (GraphPad Software, Inc., San Diego, CA). A simple descriptive statistical technique was employed to identify the characteristics of variables in the form of counts and percentages for categorical and nominal variables, while mean and standard deviations are reported for continuous variables. A chi-squared test has been used to establish a relationship between the categorized score and demographics. In addition, a general linear model univariate analysis was performed to determine the significant predictors using the main effect model. Finally, a p-value of <0.05 was used to reject the null hypothesis.

## Results

A total of 378 family primary caregivers completed the self-administered questionnaire, as shown in Table 1. The majority of them are 36-44 years old (27.2%), and 57.9% are male and have at least a bachelor’s degree (63.0%). In terms of employment, 50.8% of them are employed, 40.5% are unemployed, and only 8.7% are students. The majority of participants (37.3%) were sons/daughters of terminally ill patients, with colon cancer (25.4%) as the most prevalent diagnosis. Table 1 summarizes the demographic characteristics of the participants.

**Table 1:** Demographic characteristics of the study participants

Demographics		Count	%
Total		378	100.0
Age	18-25 years	47	12.4
	26-35 years	94	24.9
	36-44 years	103	27.2
	45-55 years	81	21.4
	56 years and more	53	14.0
Gender	Male	219	57.9
	Female	159	42.1
Level of education	Basic education (High school and lower)	140	37.0
	Bachelor’s degree and more	238	63.0
Employment status	Student	33	8.7
	Employed	192	50.8
	Unemployed	153	40.5
Relation to the patient	Father/mother	42	11.1
	Brother/sister	85	22.5
	Son/daughter	141	37.3
	Husband/wife	62	16.4
	Other	48	12.7
Duration of receiving palliative care	Less than one year	246	65.1
	More than one year	132	34.9
Tumor site	Colon	96	25.4
	Breast	63	16.7
	Lung	49	13.0
	Brain	23	6.1
	Others (genitourinary, hematology, head and neck)	147	38.9

The participants' general knowledge of PC is presented in Table 2. The majority of them (73.8%) were not aware of PC before their family member was referred to the hospital. PC is provided at specialist facilities, according to 124 out of 378 (32.8%) respondents. Moreover, 67.2% are aware that healthcare practitioners should be asked for information on PC, and 53.7% believe PC services only include analgesia and symptomatic therapy. Over half of participants (54.8%) believe PC services are provided at home. When asked which factor could improve public awareness about PC, the majority (42.9%) believe that increasing specialized PC facilities would help, and the majority of them (34.1%) believe that a lack of specialized hospitals will hinder the promotion of PC.

**Table 2:** Knowledge assessment of the study participants

Variables		Count	%
Total		378	100.0
Were you aware about palliative care before your family member was referred to this hospital for such services?	Yes	99	26.2
	No	279	73.8
Where do you think palliative care takes place?	At home	74	19.6
	At specialized facilities	124	32.8
	Both at home and at specialized facilities	116	30.7
	I do not know	64	16.9
If you needed information about palliative care, where would you look for it, or whom would you ask?^a^	Healthcare providers	254	67.2
	Internet	173	45.8
	Friends	70	18.5
What care would you expect palliative care services to deliver?^a^	Provide analgesia and symptomatic treatment	203	53.7
	Follow-up of terminally ill patients’ condition	122	32.3
	Supporting home care	143	37.8
	Health education	105	27.8
	I expect nothing from palliative services.	39	10.3
	I don't know	4	1.1
Where do you think patients receive these services?^ A^	At hospitals	131	34.7
	At home	207	54.8
	Via the phone or the internet	73	19.3
	I don't know	12	3.2
What factors would in your view promote public awareness of palliative care?^ A^	Increasing specialized facilities for palliative care	162	42.9
	Greater promotion of palliative care	150	39.7
	Improvements in palliative care	134	35.4
	I don't know	17	4.5
What factors would in your opinion hinder the promotion of public awareness of palliative care?^ A^	Lack of health education	108	28.6
	Desperate patients/caregivers	81	21.4
	Lack of specialized facilities for palliative care	129	34.1
	Lack of palliative care services	93	24.6
	I don't know	7	1.9
What could be done to promote greater openness in discussion (and to inform future strategies)?^ a^	Improving communication between caregivers and healthcare providers	127	33.6
	Providing health education about palliative care of terminally ill patients	117	31.0
	Providing more specialized healthcare facilities	149	39.4
	I don't know	1	0.3
^a^-multiple answered questions please don’t add counts and percentages.			

The findings revealed that the mean score of caregivers' general knowledge is 2.75+0.9, indicating that they have little knowledge about PC. According to Table 3, 63.5% are slightly aware of PC, 25.1% are unaware, and only 11.4% are somewhat aware.

**Table 3:** Caregivers’ general knowledge score of palliative care

Variables	N		Min	Max	Mean		SD
Caregivers’ general knowledge of palliative care	378		1.16	5.41	2.75		0.9
				Count		%	
Total				378		100.0	
Caregivers’ general knowledge of palliative care		Not Aware (<2)		95		25.1	
		Slightly Aware (<4-2)		240		63.5	
		Somewhat Aware (<6-4)		43		11.4	
		Very Aware (6-8)		0		0.0	

Table 4 shows the results regarding caregivers' general knowledge of PC in relation to their demographic characteristics. It is notable that age, employment status, patient relationship, and duration of receiving PC (all p<0.001) are all significantly associated with their knowledge. No associations were found on gender, level of education, and tumor site.

**Table 4:** Factors associated with study participants’ general knowledge of palliative care

Demographics		Total	Caregivers’ general knowledge of palliative care				p-value
			Not Aware (<2)	Slightly Aware (<4-2)	Somewhat Aware (<6-4)	Very Aware (6-8)	
Total		378	95 (25.1%)	240 (63.5%)	43 (11.4%)	0 (0.0%)	-
Age	18-25 years	47	8 (17.0%)	30 (63.8%)	9 (19.1%)	0 (0.0%)	<0.001^a^
	26-35 years	94	20 (21.3%)	53 (56.4%)	21 (22.3%)	0 (0.0%)	
	36-44 years	103	25 (24.3%)	72 (69.9%)	6 (5.8%)	0 (0.0%)	
	45-55 years	81	24 (29.6%)	50 (61.7%)	7 (8.6%)	0 (0.0%)	
	56 years and more	53	18 (34.0%)	35 (66.0%)	0 (0.0%)	0 (0.0%)	
Gender	Male	219	53 (24.2%)	143 (65.3%)	23 (10.5%)	0 (0.0%)	0.672
	Female	159	42 (26.4%)	97 (61.0%)	20 (12.6%)	0 (0.0%)	
Level of education	Basic education (high school and lower)	140	43 (30.7%)	83 (59.3%)	14 (10.0%)	0 (0.0%)	0.154
	Bachelor’s degree and more	238	52 (21.8%)	157 (66.0%)	29 (12.2%)	0 (0.0%)	
Employment status	Student	33	1 (3.0%)	24 (72.7%)	8 (24.2%)	0 (0.0%)	0.001^a^
	Employed	192	52 (27.1%)	114 (59.4%)	26 (13.5%)	0 (0.0%)	
	Unemployed	153	42 (27.5%)	102 (66.7%)	9 (5.9%)	0 (0.0%)	
Relation to the patient	Father/mother	42	5 (11.9%)	28 (66.7%)	9 (21.4%)	0 (0.0%)	<0.001^a^
	Brother/sister	85	9 (10.6%)	62 (72.9%)	14 (16.5%)	0 (0.0%)	
	Son/daughter	141	39 (27.7%)	90 (63.8%)	12 (8.5%)	0 (0.0%)	
	Husband/wife	62	26 (41.9%)	33 (53.2%)	3 (4.8%)	0 (0.0%)	
	Other	48	16 (33.3%)	27 (56.3%)	5 (10.4%)	0 (0.0%)	
Duration of receiving palliative care	Less than one year	246	43 (17.5%)	171 (69.5%)	32 (13.0%)	0 (0.0%)	<0.001^a^
	More than one year	132	52 (39.4%)	69 (52.3%)	11 (8.3%)	0 (0.0%)	
Tumor site	Colon	96	32 (33.3%)	54 (56.3%)	10 (10.4%)	0 (0.0%)	0.127
	Breast	63	15 (23.8%)	41 (65.1%)	7 (11.1%)	0 (0.0%)	
	Lung	49	6 (12.2%)	35 (71.4%)	8 (16.3%)	0 (0.0%)	
	Brain	23	2 (8.7%)	17 (73.9%)	4 (17.4%)	0 (0.0%)	
	Others	147	40 (27.2%)	93 (63.3%)	14 (9.5%)	0 (0.0%)	
^a^-significant using the chi-square test at the <0.05 level							

The general linear model at the <0.05 level showed statistical differences in participants’ knowledge by age, employment status, relation to patient, and duration of receiving PC. Participants aged from 26-35 years old (p<0.001), those who are students (p=0.002), those who are brothers/sisters of the patient (p<0.001), and those who are less than one year of PC (p<0.001) significantly related to caregivers’ knowledge (Table 5).

**Table 5:** Determination of factors associated with caregivers’ general knowledge using the general linear model

Dependent Variable: Caregivers’ general knowledge of palliative care					
Parameter	B	S.E.	95% C.I.		p-value
			Lower Bound	Upper Bound	
Intercept	1.360	0.116	1.131	1.589	<0.001^a^
Age=18-25 years	0.173	0.118	-0.060	0.406	0.145
Age=26-35 years	0.361	0.095	0.174	0.548	<0.001^a^
Age=36-44 years	0.129	0.092	-0.052	0.310	0.163
Age=45-55 years	0.027	0.097	-0.165	0.218	0.783
Employment status=Student	0.385	0.127	0.137	0.634	0.002^a^
Employment status=Employed	0.040	0.060	-0.077	0.158	0.498
Relation to the patient=Father/mother	0.237	0.124	-0.007	0.480	0.057
Relation to the patient=Brother/sister	0.356	0.101	0.158	0.554	<0.001^a^
Relation to the patient=Son/daughter	0.081	0.092	-0.101	0.263	0.380
Relation to the patient=Husband/wife	-0.041	0.110	-0.258	0.176	0.709
Duration of receiving palliative care=Less than one year	0.255	0.060	0.137	0.374	<0.001^a^
^a^-significant using general linear model at <0.05 level					

## Discussion

PC is an essential approach to providing care for patients with terminal illnesses. The World Health Organization (WHO) has suggested that PC be used in healthcare services worldwide [3]. However, the availability of PC services remains limited in various regions of the world, including Saudi Arabia [5]. One possible limitation to PC utilization is a lack of understanding and access to PC and misconceptions regarding the nature of this service [6]. According to studies, knowledge and experience are essential elements in determining attitudes and choices for obtaining care [10,13]. In this study, general knowledge of family caregivers in KAMC, Makkah, Saudi Arabia, was determined and evaluated.

Findings revealed that participants have limited knowledge of PC. A large percentage of the participants (73.8%) were unaware of PC before the patient was referred to the hospital. Similarly, the study by Chaves showed that 78% of the participants were unaware of PC [14], while 70% of the family caregivers in the study of Dionne-Odom et al. did not know PC [10]. In Trivedi et al.'s nationwide study, 70% of participants were also unaware of PC [15]. On the other hand, the percentage of unaware participants in this study was higher than that of Ibraheem et al., in which 32.3% of caregivers at Najran University Hospital were unaware of PC [7]. A possible cause may rely on the nature and background of previous exposure to PC service, which may lead to this discrepancy between this study's results and other studies.

In the present study, only age, employment status, relation to the patient, and duration of receiving PC were significantly associated with caregivers’ knowledge. Caregivers between the 26-35 age group were somewhat more aware than the other age group. In the study of Kozlov et al. in 2018, among community-dwelling adults, the 45-64 age group had a higher Palliative Care Knowledge Scale Score than another age group [16]. Similarly, in the nationwide study of Huo et al. of US adults, 50-64 and 65-year-olds have better knowledge of PC [17].

This study also found that students have greater knowledge than employees. This might be because younger individuals learn about PC in school or have better internet access. Although education is not significantly related to the knowledge of the participants in this study, previous research showed the importance of educational attainment and experience in PC [17,18]. Those who have higher educational attainment are likely to have better health knowledge, which may extend to the context of PC.

Participants in this study believed that increasing the number of specialized PC institutions would increase public awareness of PC. They further thought that the main barrier to shared knowledge is a lack of specialized facilities, followed by a lack of health education. According to Ibraheem et al., increasing societal knowledge about PC is the most important means to improve awareness, followed by expanding patients' access to palliative centers and then improving PC services [7].

The findings of the current study could be used by public health organizations to focus their efforts on increasing general public awareness and education on the nature and services of PC. Such findings are crucial for effectively increasing PC knowledge. To improve knowledge regarding PC, practical educational activities, as well as access to providers of PC, should be implemented. It is vital to enable those who are unaware and have a negative attitude toward PC to fully understand the purpose of PC and its advantages. Some studies have already reported that the successful implementation of PC necessitates considering the local resources and customs, and those educational initiatives should be observed in training healthcare providers and even volunteers [19,20].

Limitations

Despite the current study providing some significant implications, it has some limitations. One limitation is that all of the patients originate from a single hospital, which may limit the generalizability of the results. However, because the study sample is large and diverse, the results may be conclusive. Second, the findings do not appear to indicate causal correlations between factors; further research is required to investigate the cause-and-effect relationships between personal characteristics and PC knowledge to enhance a better understanding and explanation of the findings. Finally, using a self-reported questionnaire may have resulted in an overestimation or underestimation of the questions, increasing the potential for response bias.

## Conclusions

This study demonstrated the general knowledge of the family caregivers in KAMC, Makkah, Saudi Arabia. The low knowledge mean score was found to be 2.75+0.9, indicating participants' limited knowledge of PC. Findings further revealed that age, employment status, relation to the patient, and duration of receiving PC were associated with raised knowledge. This study showed that patients believed increasing specialized facilities would raise public awareness. This study recommended that effective PC education programs and more specialized facilities be implemented to enhance the awareness of both caregivers and patients.

## Appendices

Appendix 1. Interview guide

Caregivers’ general knowledge of palliative care

•    Were you aware about palliative care before your family member was referred to this hospital for such services?

•    Yes

•    No

•    Where do you think palliative care takes place?

•    At home

•    At specialized facilities

•    Both at home and at specialized facilities

•    I do not know

•    If you needed information about palliative care, where would you look for it, or whom would you ask? (participants were allowed select more than one answer)

•    Healthcare providers

•    Internet

•    Friends

•    Other, please specify

Expectations

•    What care would you expect palliative care services to deliver?

(participants were allowed select more than one answer)

•    Provide analgesia and symptomatic treatment

•    Follow-up of terminally ill patients’ condition

•    Supporting home care

•    Health education

•    I expect nothing from palliative services

•    Other, please specify

Accessibility

•    Where do you think patients receive these services?

(participants were allowed to select more than one answer)

•    At hospitals

•    At home

•    Via the phone or the internet

•    Other, please specify

Future strategies

•    What factors would in your view promote public awareness of palliative care?

(participants were allowed to select more than one answer)

•    Increasing specialized facilities for palliative care

•    Greater promotion of palliative care

•    Improvements in palliative care provided

•    Other, please specify

•    What factors would in your opinion hinder the promotion of public awareness of palliative care? (participants were allowed to select more than one answer)

•    Lack of health education

•    Desperate patients/caregivers

•    Lack of specialized facilities for palliative care

•    Lack of palliative care services

•    Other, please specify

•    What could be done to promote greater openness in discussion (and to inform future strategies)?

(participants were allowed to select more than one answer)

•    Improving communication between caregivers and healthcare providers

•    Providing health education about palliative care of terminally ill patients

•    Providing more specialized healthcare facilities • Other, please specify
